# Digital Health Literacy, Technology Acceptance, and Competence Among Older Adults Aged ≥65 Years: Cross-Sectional Study Investigating Differences Between Women and Men

**DOI:** 10.2196/85846

**Published:** 2026-05-14

**Authors:** Franziska Ulrike Jung, Melanie Luppa, Matthias Reusche, Kerstin Wirkner, Melanie Eberl, Yvonne Dietz, Christoph Engel, Steffi G Riedel-Heller

**Affiliations:** 1 Institute of Social Medicine, Occupational Health and Public Health (ISAP), Faculty of Medicine, Leipzig University, Philipp-Rosenthal-Str. 55, Leipzig, 04103, Germany, 49 03419724571; 2 Institute for Medical Informatics, Statistics and Epidemiology, Leipzig University, Leipzig, Germany; 3 LIFE—Leipzig Research Centre for Civilization Diseases, Leipzig University, Leipzig, Germany

**Keywords:** digital health literacy, technology, acceptance, competence, older adults

## Abstract

**Background:**

Digital health literacy (DHL) has the potential to improve health among older adults by enhancing access to health-related information and health care services.

**Objective:**

The aim of this study was to analyze the relationship between DHL and technology commitment in adults aged 65 years and older, while also investigating possible gender differences.

**Methods:**

The analytical sample consisted of 1824 individuals. The analysis included descriptive comparisons in terms of DHL, technology acceptance, competency, support, and internet use. Multivariate regression models (generalized linear models) were applied in order to test the association between DHL and technology commitment, controlling for internet use as well as health-related and sociodemographic characteristics.

**Results:**

Male and female participants did not differ in terms of DHL (mean score: 3.5, SD 1.2 [men] and 3.5, SD 1.3 [women]; *P*=.70); however, male participants reported significantly higher technology acceptance (*P*<.001) and higher technology competencies (*P*<.001), but less support with regard to technology use (*P*<.001). Within regression models, only higher technology acceptance (coefficient=0.023, 95% CI 0.006‐0.041; *P*=.01) and support (coefficient=0.027, 95% CI 0.014‐0.040; *P*<.001) were significantly linked to greater DHL. The subgroup analysis revealed that DHL was significantly associated with technology acceptance among men (coefficient=0.036, 95% CI 0.012‐0.060; *P*=.003) but not women (coefficient=0.024, 95% CI 0.008‐0.040; *P*=.44).

**Conclusions:**

According to the current results, DHL is highly related to technology commitment. Gender differences should be taken into account when developing and evaluating appropriate interventions to improve DHL by addressing the acceptance of technologies and optimizing support infrastructures.

## Introduction

The association between digital health literacy (DHL) and technology use or acceptance among older adults is a critical area of research, given the increasing reliance on digital platforms for health information and services. This relationship reflects how DHL informs technology acceptance, as well as technology competencies, and facilitates a greater use of health technologies.

Previous research highlights the importance of internet access and participation in order to improve health outcomes and prevent health care disparity [1,2]. A study investigating internet use in old age in 2016 has found that only 27% reported using the internet on a daily basis and 57% never used the internet [3]. However, the increasing proportion of internet users aged 65 years and older is a well-documented demographic trend [4]. Multiple studies found evidence for the gradual rise of internet engagement among the senior population, driven by factors including health-related motivations, social connectivity, and technological advancements. In this context, 81% (62/242) of internet users aged 60 years and above use the internet for health- or disease-related searches [5].

In order to successfully apply health-related information in decision-making and health behavior, it is important to understand and critically evaluate digital information and services. Although there is now a clear definition, DHL is often described as the skills required to seek, understand, and use health information from digital sources to make health-related decisions [6-8]. According to a recent systematic review, higher levels of DHL among older adults were related to being younger, a better health status, a higher socioeconomic status, higher education levels, and higher income [9]. In addition, social connectivity and participation, as well as positive attitudes toward internet and digital health information, have also been associated with higher digital literacy [9]. Especially among the older adults, age-related limitations with regard to vision or hearing may influence the motivation to adopt digital technologies and therefore influence DHL [10,11]. According to a study by Liu et al, perceived usefulness, ease of use, and reliability of digital health information may not only impact the DHL of older adults. Those who view these factors positively are also more likely to use digital health services or devices [12]. This relationship is particularly rooted in the technology acceptance model (TAM) [13], which posits that users’ attitudes toward technology may stem from perceived attributes and personal needs [12,14]. Similarly, Wei and Zhou [15] found that higher DHL correlates with more frequent engagement in health-promoting activities, suggesting that improved literacy not only facilitates the use of technology but also leads to effective integration into health management practices.

Moreover, the willingness of older adults to engage with digital health resources has been shown to be influenced by the degree of technology acceptance or commitment. Kim et al [16] reported that older adults’ intentions to use digital health applications are significantly shaped by their attitudes toward new technologies, arguing that negative perceptions may hinder technology adoption. The fear of using complex technologies often leads to resistance, compounded by anxiety about using these tools. In this context, higher anxiety levels correlated with lower eHealth literacy, as indicated by Kim et al [16]. These findings align with those of Rasekaba et al [17], who highlighted the impact of low digital literacy on older adults’ ability to engage with such technologies due to less confidence and limited access, consequently creating a digital divide that can affect their health management. However, technology acceptance, competencies, and support are understudied mechanisms, which link technology and DHL in older adults. While technology use and acceptance are often treated as prerequisites for digital engagement, DHL represents a distinct, domain-specific construct that extends beyond general digital skills or device use. DHL requires the ability to locate, critically evaluate, and apply health information in digital environments—competencies that are not necessarily captured by measures of technology adoption or acceptance alone.

The exploration of gender-specific findings in the context of DHL and technology acceptance among older adults reveals several nuances that warrant consideration. Research indicates that gender differences exist, influencing both levels of DHL and the willingness to engage with technology. According to a recent review, older women had lower eHealth literacy compared to older men [18]. However, the authors suggest that the gender-related disparity may gradually diminish and, in addition, may be explained by differences regarding (lower) socioeconomic status. This emphasizes the complexity of social determinants affecting DHL and suggests that factors such as income, education, or social support may also be important, by influencing access to digital health resources [19].

The aim of this study was to investigate the relationship between DHL and technology use and acceptance among individuals aged 65 years and older. Importantly, rather than assuming gender differences in DHL itself, this study investigates whether the factors associated with DHL differ by gender, thereby addressing inconsistencies in the existing literature on adults aged 65 years and older. Based on previous studies, we hypothesize that a higher degree of acceptance and higher competencies as well as support in relation to technology use may be related to higher DHL. Furthermore, we aim to explore possible gender differences using the subgroup analysis. In this context, the impact of sociodemographic characteristics will be taken into account.

## Methods

### Recruitment

The current data analysis is based on a cross-sectional study on DHL among old and oldest people in Saxony (Germany). For the purpose of recruitment, 3000 participants from the participant pool of the LIFE (Leipzig Research Centre for Civilization Diseases) Adult Cohort were randomly chosen, recontacted, and surveyed using a paper-and-pencil questionnaire. Overall, a sample of 2227 returned the questionnaire, representing a response rate of 74.2%. The only inclusion criteria were being at least 65 years old at the time of the survey (May 2024). The study and recruitment procedure of the overall LIFE Adult Study has been already provided elsewhere [20,21]. The LIFE Adult Study is a population-based cohort study and includes data of 10,000 randomly selected participants from the City of Leipzig (Germany). The objective of the overall LIFE Adult Study is to investigate prevalences, predispositions, and determining factors of major civilization diseases over time.

### Ethical Considerations

The LIFE Adult Study was conducted in accordance with the Declaration of Helsinki, and ethical approval was granted by the Ethics Committee of the Medical Faculty of Leipzig University (approval numbers 263–2009‐14122009, 263/09-ff, 201/17-ek). Each participant provided written informed consent. At the start of the survey, all participants were informed about the aim of the study, as well as data handling and protection. Participation was anonymous and voluntary, and withdrawal of consent was possible at any time during the study. Participants did not receive incentives for participating in this study. The data privacy and confidentiality was approved by the responsible data protection officer.

### Study Design

Apart from sociodemographic and health-related information (ie, age, sex, income, and general health status) and DHL, the questionnaire assessed internet use, technology acceptance, and competency. The instruments and constructs are described in more detail as follows:

DHL was investigated using the 8-item revised German eHealth Literacy Scale (GR-eHEALS) [22]. Exploratory and confirmatory factor analyses in previous studies confirmed the validity and factorial structure of the GR-eHEALS [22,23]. The items have been described in greater detail elsewhere [22]. DHL is measured using a 5-point Likert scale ranging from 1 (*do not agree at all*) to 5 (*do fully agree*), with higher scores indicating greater DHL (Cronbach α 0.970). The scale consists of 4 items that assess how information is searched on the internet (example item: “I know where I can find helpful health information on the internet”). The other 4 items focus on how the information found may be evaluated (example item: “I can distinguish between trustworthy and dubious websites with health information”).

Eight items of the short scale on technology commitment were used to assess technology acceptance and self-perceived competence on a 5-point Likert scale [24]. A translation of the items and scale can be found in Multimedia Appendix 1. In this study, Cronbach α was 0.895 for the subscale self-perceived technology competence and 0.927 for the subscale technology acceptance. The scale has been used widely and validated among older age groups [25,26]. Furthermore, 3 items were used to assess social support in relation to technology use [27]. The items and the answer options can be found in Multimedia Appendix 1. Cronbach α was 0.954.

In order to survey internet usage, the following items were included in the survey: “how often they were using the internet?” (ranging from 0*=*never to 7*=*several times a day); “how familiar they are with using the internet” (ranging from 0*=*not very familiar to 3*=*very familiar); and “how often do you use the internet to search for information that may affect your health” (ranging from 0*=*never to 4*=*always) for both items. These items have been used in similar studies before [28].

In order to survey the sex, the answer categories, male or female, were provided. Educational attainment was assessed by sociodemographic items on the highest school qualification and highest job training qualification, resulting in low, intermediate, or high educational attainment, known as CASMIN (Comparative Analysis of Social Mobility in Industrial Nations) categories. The procedure and calculation of the CASMIN categories have been described elsewhere [29,30]. Family status was surveyed using the following 5 categories: single, married, divorced, widowed, and married, but separated. Household net income (monthly) was assessed using the following answer categories: <€1000, €1000 to <€1500, €1500 to <€2000, €2000 to <€2500, €2500 to <€3000, €3000 to <€3500, and more than €3500.

Perceived health status was surveyed using the EuroQol Visual Analogue Scale. Participants were asked to rate their current health status on a scale between 0 (*worst health status*) and 100 (*best health status*) [31]. In addition, participants were asked to rate their eyesight using the following question: “Do you have trouble seeing?” Possible answers included no impairment, mild impairment, significant impairment, and most serious impairment [32]. Similarly, they were asked to rate their hearing skills using the following item: “Do you have any trouble hearing?” (answer categories: no impairment, mild hearing loss, significant hearing loss, and extreme hearing loss/deafness) [32].

Individual competence expectations when dealing with difficulties in everyday life settings (self-efficacy) were measured using the ASKU (Allgemeine Selbstwirksamkeit Kurzskala) scale [33]. The instrument consists of 3 items and can be answered using a 5-point Likert scale. The items can be found in Multimedia Appendix 1. Cronbach α was 0.886.

### Statistical Analysis

Overall, 3000 individuals were contacted by mail in which 2227 individuals replied. After removing cases with missing information, the analytical sample contained 1824 individuals. The analytical sample was significantly younger (*P*<.001; 75.3 y vs 77.9 y) and more highly educated (*P*<.001). However, no significant differences were found with regard to sex.

During a first step, differences between male and female participants were analyzed descriptively and compared using *χ*² tests, *t* tests, and Kruskal-Wallis tests, as appropriate. In addition, multivariate regression models were applied to investigate the relationship between DHL (outcome variable, continuous) and technology commitment and support (predictor variables) across the overall sample (model 1). Before the analysis, we assessed multicollinearity and tested for normality. No multicollinearity between variables was found. Due to the skewness of the outcome variable (DHL), generalized linear models with robust variance estimators were calculated (γ family, link-log [34]). Generalized linear models—regression models with γ distribution—are widely used in modeling continuous, nonnegative, and positive-skewed data. The subgroup analysis was conducted to investigate possible sex-related differences (model 2: female subsample; model 3: male subsample). All regression models were controlled for sociodemographic characteristics, relevant health parameters (health status and eyesight), self-efficacy, and aspects related to internet use. Covariates were chosen based on relevance for DHL or the relationship between DHL and internet use or technology commitment. Statistical analyses were performed using the software package STATA, version 16 (StataCorp), assuming an α level of .05 (2-tailed) as an indicator for statistical significance.

## Results

### Participants Characteristics

Overall, the majority in this sample (N=1824) was female (n=1016, 53%) and married (n=1247, 65%), and the mean age of the participants was 75 (SD 6.4) years. Further sociodemographic characteristics and differences between female and male participants are summarized in Table 1. In this study, men and women differed in terms of marital status (*P*<.001), education (*P*<.001), income (*P*<.001), and overall health status (*P*=.02). No significant differences were found with regard to general self-efficacy. No significant differences between female and male individuals were found in terms of DHL. However, men reported using the internet more frequently and feeling more confident while using the internet (*P*<.001 for both).

**Table 1.** Characteristics of the overall study sample, the female subsample, and the male subsample (N=1824).

Characteristics	Overall (N=1824)	Women (n=973)	Men (n=851)	Significance (*P* value)
Age (y), mean (SD; range)	75.3 (6.4; 65‐91)	74.7 (6.3; 65‐91)	75.9 (6.4; 65‐90)	<.001
Marital status, n (%)				<.001
Married	1.191 (65.3)	544 (56)	647 (76)	
Married, but separated	35 (1.9)	13 (1.3)	22 (2.6)	
Single	71 (3.9)	34 (3.5)	37 (4.3)	
Divorced	213 (11.7)	136 (14)	77 (9.1)	
Widowed	314 (17.2)	246 (25.3)	68 (8)	
Education (CASMIN^a^), n (%)				<.001
Low	187 (10.2)	103 (10.6)	84 (9.8)	
Intermediate	799 (43.9)	503 (51.8)	296 (34.9)	
High	838 (45.9)	367 (37.7)	471 (55.3)	
Household income (€), monthly, n (%)				<.001
<1499	206 (11.3)	125 (12.9)	81 (9.5)	
1500‐1999	252 (13.8)	169 (17.4)	83 (9.8)	
2000‐2499	396 (21.7)	224 (23)	172 (20.1)	
2500‐2999	352 (19.3)	169 (17.4)	183 (21.6)	
3000‐3499	294 (16.1)	144 (14.8)	150 (17.7)	
>3500	324 (17.7)	142 (14.6)	182 (21.3)	
Overall health status^b^, mean (SD)	69.4 (18.6)	70.3 (18.4)	68.3 (18.8)	.02
Eyesight, n (%)				.67
No impairment	1.564 (85.7)	831 (85.3)	733 (86.2)	
Mild impairment	221 (12.1)	118 (12.1)	103 (12.1)	
Significant impairment	37 (2.1)	23 (2.5)	14 (1.6)	
Most serious impairment	2 (0.1)	1 (0.1)	1 (0.1)	
Hearing, n (%)				—^c^
No impairment	1.247 (68.4)	719 (73.9)	528 (62)	
Mild hearing loss	515 (28.2)	224 (23)	291 (34.2)	
Significant hearing loss	58 (3.2)	27 (2.8)	31 (3.6)	
Extreme hearing loss/deafness	4 (0.2)	3 (0.3)	1 (0.1)	
Digital health literacy^d^, mean (SD)	3.5 (1.2)	3.5 (1.3)	3.5 (1.2)	.70
Self-efficacy^e^, mean (SD)	12.4 (2.1)	12.4 (2.1)	12.4 (2.2)	.80
Internet use				
Frequency^f^, mean (SD)	5.2 (2.4)	5 (2.5)	5.5 (2.3)	<.001
Confidence^g^, mean (SD)	1.7 (0.9)	1.6 (0.9)	1.8 (0.9)	<.001
Health-related^h^, mean (SD)	1.6 (1.2)	1.6 (1.2)	1.6 (1.1)	.05

a CASMIN: Comparative Analysis of Social Mobility in Industrial Nations.

b A higher score indicates better overall health, range: 0-100.

c Not available.

d A higher score indicates greater digital health literacy, range: 2-10.

e A higher score indicates greater self-efficacy, range: 3-15.

f A higher score indicates more frequent use, range: 0-7.

g A higher score indicates feeling more familiar using the internet, range: 0-3.

h A higher score indicates greater health-related internet use, range: 0-4.

**Figure 1.**
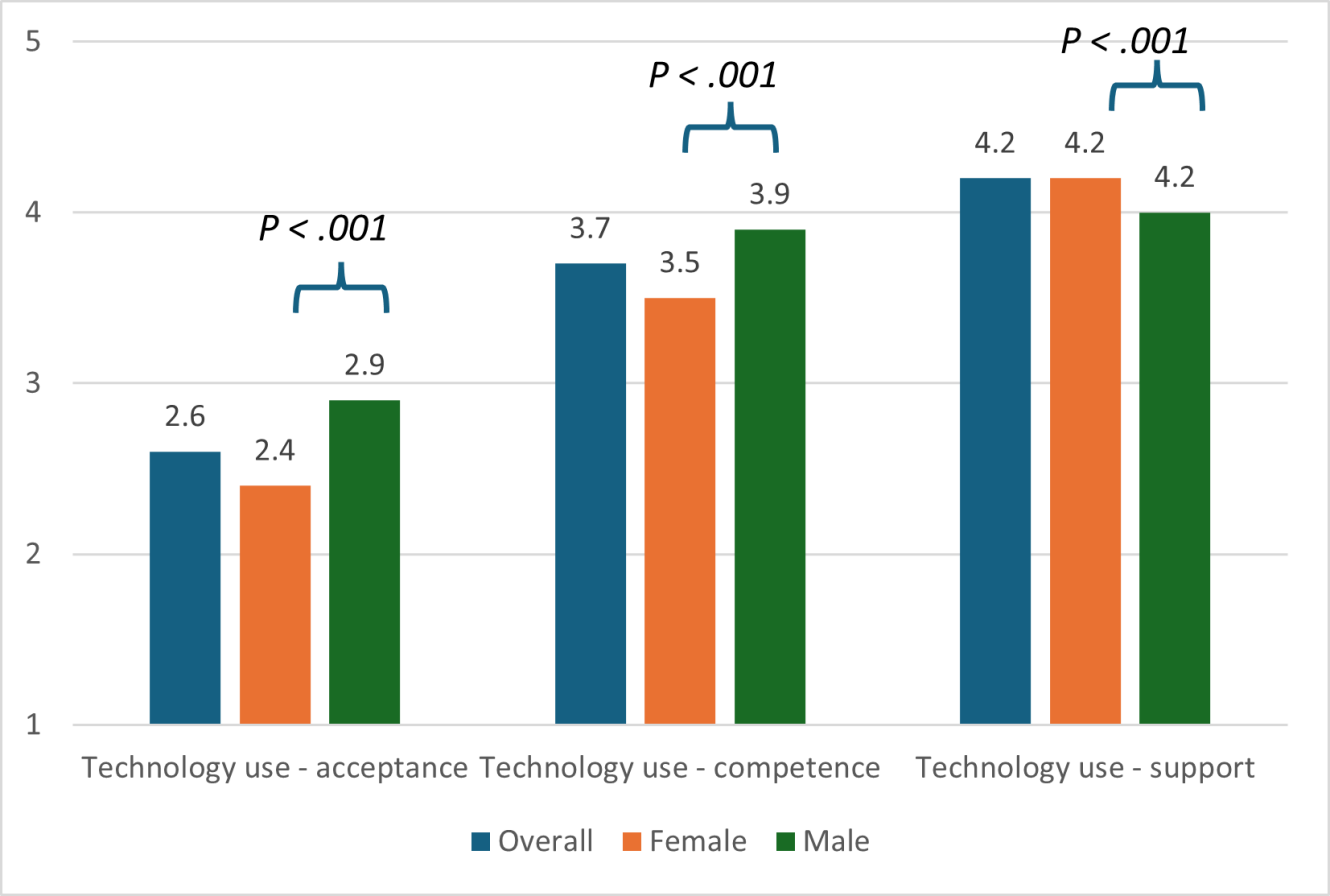
Characteristics of technology use among the older adults (overall, women, and men, n=1824). Acceptance: the higher score indicates greater acceptance of technology; competence: the higher score indicates higher self-perceived competence with regard to technology use; support: the higher score indicates greater support with regard to technology use.

Characteristics of technology use in terms of acceptance, competence, and social support have also been analyzed (Figure 1). In this context, male participants reported higher technology acceptance (*P*=.001), higher technology competencies (*P*<.001), but less support with regard to technology use (*P*<.001; Table 1).

### General Regression Analysis

In addition to descriptive analysis, regression analysis was conducted. Table 2 shows the results of the multivariate generalized regression model with DHL as the outcome variable (model 1). When controlling for sociodemographic and health-related variables, higher technology acceptance (coefficient=0.023, *P*=.01) and support were linked to greater DHL (coefficient=0.027, *P*<.001), indicating that motivational readiness and access to assistance play an important role in older adults’ ability to navigate digital health information. Similarly, feeling more confident and greater frequency of internet use (in general and with regard to health-related aspects) were also significantly linked to better DHL (*P*<.001 for all). Interestingly, self-perceived technology competence was not related to DHL (coefficient=0.005, *P*=.61). This finding suggests that the self-assessment of overall technology competence captures a broader construct that may not be directly related to health-specific digital literacy skills within this study population. Taken together, the included predictors explained a substantial proportion of the variance in DHL (61% of 100% variance explained), underscoring the importance of motivational, experiential, and support-related factors beyond sociodemographic characteristics alone.

**Table 2.** Regression analysis with digital health literacy as the outcome and internet use and technology affinity as predictor variables, controlling for sociodemographic characteristics and health-related factors (model 1^a^, N=1824).

Variables	Coefficient (95% CI)	*P* value
Internet use and technology affinity		
Technology use		
Acceptance	0.023 (0.006 to 0.041)	.01
Competence	0.005 (−0.014 to 0.024)	.61
Support	0.027 (0.014 to 0.040)	<.001
Internet use		
Confidence	0.153 (0.125 to 0.182)	<.001
Frequency	0.057 (0.046 to 0.069)	<.001
Health-related search	0.080 (0.067 to 0.094)	<.001
Health and self-efficacy		
Self-efficacy	0.016 (0.008 to 0.024)	<.001
Overall health	0.001 (−0.001 to 0.002)	.48
Eyesight (reference: no impairment), *χ*^2^ (*df*)=6.69 (3), *P*=.08		
Mild impairment	−0.022 (−0.062 to 0.019)	.29
Significant impairment	−0.090 (−0.204 to 0.024)	.12
Most serious impairment	−0.278 (−0.561 to 0.006)	.10
Hearing (reference: no impairment), *χ*^2^ (*df*)=1.97 (3), *P*=.58		
Mild hearing loss	−0.019 (−0.050 to 0.012)	.22
Significant hearing loss	0.009 (−0.075 to 0.093)	.83
Extreme hearing loss/deafness	0.110 (−0.287 to 0.507)	.59
Sociodemographic characteristics		
Age	0.001 (−0.001 to 0.003)	.44
Sex (reference: male)	0.029 (0.001 to 0.057)	.045
Marital status (reference: married), *χ*^2^ (*df*)=6.54 (4), *P*=.16		
Married, but separated	−0.018 (−0.125 to 0.089)	.74
Single	−0.055 (−0.123 to 0.013)	.11
Divorced	−0.009 (−0.051 to 0.034)	.70
Widowed	−0.047 (−0.090 to −0.005)	.03
Education^b^ (reference: low), *χ*^2^ (*df*)=3.70 (2), *P*=.16		
Mediate	0.040 (−0.023 to 0.102)	.22
High	0.016 (−0.046 to 0.078)	.61
Income	−0.008 (−0.017 to 0.002)	.11

a *R*2=0.610.

b Comparative Analysis of Social Mobility in Industrial Nations (CASMIN).

### Regression Analysis With Regard to Gender

Gender-stratified analyses revealed both shared and gender-specific patterns in the factors associated with DHL. The results can be found in Table 3, which shows that DHL was significantly associated with technology acceptance (coefficient=0.036, *P*=.003) among men (model 3) but not among women (model 2), suggesting that motivational readiness may play a more prominent role in shaping DHL for older men. The other aspects related to technology and internet use, such as frequency of use and received support, appear to be relevant to both genders, as indicated by *P* levels <.001. In other words, experiential use and access to assistance represent common enabling factors across genders. Additionally, 61% of DHL could be explained by our set of predictors.

**Table 3.** Subgroup analysis: regression analysis with digital health literacy as the outcome and internet use and technology affinity as predictor variables, controlling for sociodemographic characteristics and health-related factors (model 2: female subsample; model 3: male subsample).

Variables	Model 2^a^: women (n=973)		Model 3^b^: men (n=851)	
	Coefficient (95% CI)	*P* value	Coefficient (95% CI)	*P* value
Internet use and technology affinity				
Technology use				
Acceptance	0.010 (−0.015 to 0.035)	.44	0.036 (0.012 to 0.060)	.003
Competence	0.004 (−0.022 to 0.029)	.78	0.004 (0.025 to 0.033)	.79
Support	0.028 (0.009 to 0.048)	.005	0.024 (0.008 to 0.040)	.003
Internet use				
Confidence	0.198 (0.155 to 0.242)	<.001	0.112 (0.075 to 0.150)	<.001
Frequency	0.048 (0.032 to 0.063)	<.001	0.069 (0.052 to 0.085)	<.001
Health-related search	0.071 (0.051 to 0.091)	<.001	0.086 (0.069 to 0.104)	<.001
Health and self-efficacy				
Self-efficacy	0.012 (0.001 to 0.024)	.03	0.018 (0.006 to 0.029)	.002
Overall health	4.2×10^5^ (−0.001 to 0.001)	.95	0.001 (−0.001 to 0.002)	.39
Eyesight (reference: no impairment), model 2: *χ*^2^ (*df*)=5.61 (3), *P*=.13; model 3: *χ*^2^ (*df*)=30.30 (3), *P*<.001				
Mild impairment	−0.016 (−0.077 to 0.046)	.62	−0.027 (−0.079 to 0.025)	.31
Significant impairment	−0.145 (−0.305 to 0.014)	.07	0.001 (−0.150 to 0.152)	.99
Most serious impairment	−0.061 (−0.129 to 0.006)	.08	−0.498 (−0.676 to −0.320)	<.001
Hearing (reference: no impairment), model 2: *χ*^2^ (*df*)=2.74 (3), *P*=.43; model 3: *χ*^2^ (*df*)=47.26 (3), *P*<.001				
Mild hearing loss	−0.016 (−0.065 to 0.033)	.53	−0.025 (−0.064 to 0.014)	.21
Significant hearing loss	0.041 (−0.105 to 0.188)	.58	−0.023 (−0.115 to 0.070)	.63
Extreme hearing loss/deafness	0.272 (−0.109 to 0.653)	.16	−0.449 (−0.580 to −0.319)	<.001
Sociodemographic characteristics				
Age	−0.001 (−0.005 to 0.003)	.65	0.003 (−0.001 to 0.006)	.12
Marital status (reference: married), model 2: *χ*^2^ (*df*)=1.15 (4), *P*=.89; model 3: *χ*^2^ (*df*)=7.23 (4), *P*=.12				
Married, but separated	−0.004 (−0.175 to 0.168)	.97	−0.015 (−0.149 to 0.118)	.82
Single	−0.018 (−0.136 to 0.100)	.77	−0.075 (−0.154 to 0.005)	.07
Divorced	−0.003 (−0.058 to 0.063)	.93	0.007 (−0.065 to 0.052)	.82
Widowed	−0.026 (−0.081 to 0.280)	.34	−0.074 (−0.144 to −0.004)	.04
Education^c^ (reference: low), model 2: *χ*^2^ (*df*)=2.78 (2), *P*=.25; model 3: *χ*^2^ (*df*)=1.76 (2), *P*=.41				
Mediate	0.062 (−0.028 to 0.152)	.18	0.030 (−0.055 to 0.115)	.48
High	0.040 (−0.052 to 0.132)	.40	0.004 (−0.076 to 0.084)	.92
Income	−0.007 (−0.022 to 0.007)	.31	−0.006 (−0.018 to 0.007)	.37

a *R*2=0.609.

b *R*2=0.614.

c Comparative Analysis of Social Mobility in Industrial Nations (CASMIN).

## Discussion

### Main Findings

The aim of this study was to shed light on technology commitment and support among individuals aged 65 years and older. Moreover, we investigated the relationship between DHL and several aspects related to technology and internet use, as well as how this association may differ between men and women.

According to our results, DHL was slightly lower than other studies, but similar to studies focusing on individuals aged 65 years and older, indicating medium levels of DHL and generally lower DHL scores compared to younger individuals [35-37]. Moreover, there were no significant differences between male and female participants, aligning with another study that did not reveal any significant gender differences in eHealth literacy levels [38,39]. In contrast, it has also been argued by a recent meta-analysis that women may exhibit lower DHL than men [18]. This inconsistency emphasizes the complexity of social determinants affecting DHL and suggests that factors such as socioeconomic status or geographical location might interact with gender, influencing access to digital health resources [19]. Therefore, future studies may want to investigate whether gender differences in terms of DHL may be mediated by loneliness and living situation (in terms of social network) or whether they depend on anxiety, social pressure, and perceived risks associated with digital health services or platforms, thereby explaining if gender differences may sometimes be shaded by the impact of other factors.

In terms of technological commitment, technology acceptance scores were slightly different compared to other studies with individuals aged 60 years and older, whereas technology competence was similar to previous findings [25,26,40]. However, this study replicates previous findings that men exhibit higher technological acceptance and competence [26]. Interestingly, our findings also show that women may receive more support with regard to technology use. Our finding that significant differences were found regarding technology acceptance could be meaningfully explained by the TAM. According to TAM, technology use is not primarily determined by competencies and skills but may rather depend on perceived usefulness as well as perceived ease of use [13,41]. In other words, similar DHL levels between men and women suggest similar competencies with regard to accessing, understanding, and using health information, whereas gender differences in terms of technology acceptance reflect gender-related differences in social dynamics (an important mediator in TAM). In general, older adults often rely on social support from family members or relatives when engaging with digital technologies [27], and studies suggest that such support mechanisms may differ between men and women. Previous research on social networks and relationships has shown that women are increasingly embedded socially [27,42] and may therefore be more likely to interact with friends or community members than men when it comes to technology issues or seeking advice and assistance, thereby receiving more support. In addition, older men are often expected to be more “technically competent” due to lifelong gendered stereotypes around technology, which may lead relatives to assume they *don’t need help* [43,44].

Based on the regression analysis, we further aimed to investigate the link between DHL and technology commitment and support, controlling for sociodemographic and health-related variables. Among the overall sample, both higher technology acceptance and support were positively linked to greater DHL, confirming our prior hypotheses. Moreover, feeling more confident and frequency of internet use (general and health-related) were significantly linked to greater DHL. However, self-perceived technology competence was not significantly linked to DHL. It may be that technology acceptance and support may be stronger predictors of DHL because it promotes active engagement with digital tools and therefore motivates to learn and develop new skills, increasing DHL [45]. In contrast, self-perceived technology competence only captures a subset of abilities to develop better DHL. In other words, being able to use technical devices is a general technological competence and may not be needed for all facets of DHL (ie, being able to understand or interpret digital health information does not need technical skills). Moreover, attitudes toward technology and actual engagement—elements captured by technology acceptance and frequency of use—are supposed to facilitate DHL by promoting active interaction with digital health resources, consistent with models of technology use that highlight the relevance of behavioral engagement and perceived usefulness [13,41,46].

Interestingly, the subgroup regression analysis regarding gender revealed some differences between men and women. In other words, being interested in using new technological developments (ie, technology acceptance) was associated with greater DHL among men. These significant associations were not found among women. Men may be more likely to approach new technology through independent exploration, meaning that their curiosity or interest translates into actual learning and skill-building, hence greater DHL. Women, by contrast, may use collaborative or supported strategies to overcome barriers, which dilute the direct effect of curiosity on literacy. A study by Kim et al [16] found a connection between gender and digital literacy, revealing that older women often encounter more significant challenges and demonstrate lower self-confidence in using technology compared to their male counterparts. These disparities in digital literacy can hinder women’s ability to effectively utilize digital health resources. Therefore, gender-responsive interventions to enhance digital literacy among older females could positively impact health management outcomes [16]. However, the frequency of internet use, feeling competent and confident in using technology, and having people around who can support with technical problems were associated with greater DHL among both men and women.

According to previous and current findings, it may be a complex interplay of sociodemographic characteristics, internet use and technology commitment, and health literacy that affects older adults’ participation in digital health initiatives. Tailoring efforts to promote DHL for both genders, while considering the unique barriers faced by the older adults, could bridge the gap and lead to improved health outcomes in individuals aged 65 years and older. Importantly, the impact of improving DHL extends beyond immediate technology use; it enhances overall life satisfaction and quality of life among older adults, as indicated by studies illustrating the mediating effect of social capital in this relationship [47,48]. Increasing digital literacy may empower older individuals to take more proactive roles in managing their health, fostering a greater sense of agency and improving overall health outcomes. Future research could further investigate whether DHL is related to utilization of care, as it has been shown that general health literacy has been found to be associated with satisfaction with health care and unmet needs regarding various aspects of care [49]. Although this study did not differentiate between specific digital health devices, previous studies indicate that older adults’ engagement with digital technologies increasingly involves activities, such as searching for health- or disease-related information, accessing electronic health records, using appointment systems, and, to a lesser extent, mobile health applications for self-management or monitoring. Technology acceptance in this context may therefore be understood as being open toward engaging with different kinds of digital health content, which require not only possibilities to access but also the ability to understand and interpret electronic health information. Prospective studies could go further by investigating the acceptance of everyday digital health activities that are increasingly relevant for older adults. These may include the use of smartphones or tablets to search for health information, use patient portals, schedule appointments using web-based tools, communicate with health care providers, or use health-related applications for medication management and monitoring.

### Limitations

The aim of this study was to investigate the relationship between DHL and internet use and technology affinity. The study has some methodological constraints. Due to the cross-sectional characteristics of the study, it is not possible to draw a conclusion with regard to the direction of this relationship. In other words, better technology competence and acceptance as well as greater frequency of internet use—especially with regard to health-related aspects—may help to improve DHL. On the other hand, greater DHL due to other factors, such as generally higher self-efficacy, may lead to individuals feeling more prepared to use the internet or feel more confident, therefore using the internet more often. This, in turn, may increase opportunities for learning how to find health-related information more effectively and efficiently, therefore increasing DHL. Furthermore, the study is based on self-reported information. Participants may overestimate or underestimate their own abilities regarding (health-related) internet use. In fact, women have been found to be harder on themselves with regard to self-perceived technology capabilities, even if this does not match with their actual performance [50]. In addition, there are sample-specific issues that should be taken into account when interpreting the results. Although a heterogeneous sample in terms of education and income was analyzed, participants from rural areas or participants not living in their own (ie, living in nursing homes) may be underrepresented in this study. In this context, these individuals may experience difficulties with regard to internet access, lack possibilities to receive support in terms of technology use, or are generally more vulnerable to digital exclusion. Therefore, future studies may want to expand their sample and take into account individual living situations to further investigate possible interactions. Unfortunately, no nonresponder analysis in terms of reasons for nonparticipation could be conducted due to privacy restrictions and anonymization procedures. However, compared to similar studies, the response rate was very high (74.2%). In addition, we conducted case-wise deletion; therefore, possible selection effects should be considered regarding the current findings.

### Implication and Conclusion

The interplay between DHL and technology acceptance among older adults highlights the urgent need for focused educational interventions and supportive frameworks that facilitate the adoption of digital health technologies. Enhancing DHL may not only promote the acceptance of new technologies but also potentially transform the health management landscape for older individuals. The current results highlight the importance of individual interventions that take into account specific gender differences. Future studies may want to investigate what form of support older people desire, what needs they have (taking into account individual resources and health status), and how certain fears in using digital devices or digital sources can be addressed to improve DHL, prevent the digital divide, and reduce (age-related) health care disparities. The importance of targeted interventions is clear; effective training programs must address these gender differences to ensure that both men and women benefit equally from advancements in digital health technologies. Tailored programs may need to address specific fears and anxieties related to technology use in older women, as highlighted by Kim et al [16], which focus on technology-use anxiety. Moreover, qualitative insights from Leong et al [51] indicated that older adults’ trust in technology varies by gender and can be influenced by their experiences and their family’s input. Women, who may be more cautious toward adopting new technologies, often require validation from trusted sources before engaging [51]. In fact, prior research suggested that community or peer learning, as well as intergenerational learning, may have positive long-term effects on DHL, as well as overall health status and management among older adults [18,52,53].

## Supplementary material

10.2196/85846 Multimedia Appendix 1 Instruments.

## Abbreviations

CASMIN: Comparative Analysis of Social Mobility in Industrial Nations
DHL: digital health literacy
GR-eHEALS: German eHealth Literacy Scale
LIFE: Leipzig Research Centre for Civilization Diseases
TAM: technology acceptance model
