# Supplementary material for: Digital Health Literacy, Technology Acceptance, and Competence Among Older Adults Aged ≥65 Years: Cross-Sectional Study Investigating Differences Between Women and Men
Source: J Med Internet Res. 2026 May 14;28:e85846. doi: 10.2196/85846 (PMC13177491; doi:10.2196/85846)
Supplement: Multimedia Appendix 1 [file jmir-v28-e85846-s001.docx]

**Appendix**

Technology acceptance (Neyer et al., 2016)^1^

1. I'm very curious about new technological developments.
2. I quickly become interested in new technological developments.
3. I'm always interested in using the latest technological devices.
4. If I had the opportunity, I would use technological products much more often than I currently do.

Answering scheme: *1=not true at all* to *5=completely true*

Technology competence (Neyer et al., 2016)^1^

1. When dealing with modern technology, I'm often afraid of failure.
2. I find dealing with technological innovations overwhelming.
3. I'm afraid I'll break new technological developments rather than use them properly.
4. I find dealing with new technology difficult – I'm usually just unable to do it.

Answering scheme: *1=not true at all* to *5=completely true*

Social Support in relation to technology use (Kamin et al., 2020)

1. “I have people whom I can rely on when I have questions about technology”
2. “There are people who provide help in case of issues with technology when I need it”
3. “There are people who provide technological advice when buying new devices”.

Answering scheme: *1=do not agree* to *5=absolutely agree*

Self-efficacy (ASKU scale, Beierlein et al., 2014)

1. I can rely on my own abilities in difficult situations.
2. I am able to solve most problems on my own.
3. I can usually solve even challenging and complex tasks well.

Answering scheme: *1= does not apply at all* to *5= does fully apply*

^1^the items were translated by the authors. The original items can be found here: <https://doi.org/10.6102/zis244>
